# Epidemic Disease—Health of Buffalo

**Published:** 1866-08

**Authors:** 


### Editorial Department.

### Epidemic Disease—Health of Buffalo.

We have already reached the zenith of the hot season, and thus far our country has been free, or almost free from any epidemic disease. In New York, Brooklyn, Cincinnati, New Orleans, and perhaps some other cities, cholera is reported in sporadic form, with as yet no manifest tendency to prevail epidemically; the cases have not been sufficiently numerous to notably increase the usual death rate, and the victims are from the lowest classes. The various theories concerning it, it is needless to say, have not ALL been sustained by experience and observation thus far, and we may perhaps be justified in concluding that the circumstances and facts connected with its outbreak, throw no new light upon its mode of communication or causes, but rather teach us, that much concerning it is still unknown. The questions concerning the value of quarantine, and its ability to prevent its spread in the large cities are being answered; many conclusions will be obtainable from passing events, and some of us must conform our opinions to discovered truths. If cholera prevails to any extent, it is to be hoped that we shall observe it so impartially and closely, as to learn if possible its causes and modes of propagation, together with best means of arrest, prevention and cure.

In Buffalo the disease has not yet made its appearance in a single instance, though the public are expecting it so confidently that every case of diarrhœa, if attended, as it often is, with vomiting, is feared as cholera, to the great disadvantage of the people and to the increased labor of the physician. Diarrhœa and cholera morbus have been much more prevalent for the last few weeks, previous to which, a time of more universal good health was never enjoyed in Buffalo. We are often asked, have you seen any cholera? the manner indicating that they think if we have not, we are a little old fashioned and might have seen hundreds of cases if we had only had our eyes open to see them. An active irregular, has treated "several hundred cases successfully," and "has new ones daily;" his peculiar manner of treating cholera is infallible, (?) "not a single case has as yet proved fatal." Neighborhoods are annoyed and frightened by the frequent announcement of the outbreak of cholera, but from the best sources of information it is certain that not a single true case has yet appeared. It is greatly to be desired that physicians speak this fact positively, and not allow families to think they have been suffering from cholera, when in truth it was nothing more than the ordinary cholera morbus which is always common at this season.

There is an idea which, in most of the discussions of cholera, is not made to appear; the analogy it bears to a common and almost universal disease of the warm season in all countries, cholera morbus, as it is termed. Almost all recent writers go upon the presumption that cholera must have some definite, fixed, exclusive, peculiar and as yet undiscovered cause, which is in operation during its prevalence, and which when carefully studied, may possibly be demonstrated as portable, tangible, controllable and preventable. It is remarkable how nearly in all respects cholera morbus imitates cholera, and how the severer forms of it during prevalence of cholera, are constantly regarded both by physicians and the public as cholera. This disease springs up during the hot season, and is never wholly absent during the summer months—springs up from "common causes," or certainly arises from no specific, portable, controllable or preventable influence. During the prevalence of cholera, cholera morbus is lost—merged in that disease, indicating that these two diseases are related to each other, and that what is known of the causes of the milder disease, may help to determine also the causes of the more aggravated malady. Now it would appear quite reasonable to conclude that two diseases which resemble each other in every respect so closely, are really dependent upon a common cause, the one disease augmented in severity and rendered fatal by atmospheric influences, which, though not discoverable, are yet present and potent for evil. There are not greater differences in the manifestations and terminations of these two diseases than is observed in the eruptive fevers. Scarlet fever in its mildest form is harmless, and can scarcely be called disease, while in epidemic form it is often fatal as the cholera or plague; still other diseases manifest the same differences in malignancy and fatality between sporadic and epidemic forms. After all this, it must be confessed that cholera has features of its own which distinguish it from other forms of disease of the same general class, and that however much we may philosophize upon its character and relationships, the main questions concerning it remain still unsettled, and we must return to first principles and observe the circumstances and history of its appearance and progress, before determining its causes and modes of communication.

Cholera is reported to have made its appearance in many of the large cities simultaneously, and we have heard no intimation that it can be shown with reasonable degree of probability that it was carried in any way from one place to another—though possibly a full history may show it to have beeu so. It was developed upon the appearance of the warm season, and as yet we are hapy to believe manifests no tendency to spread by contagion or otherwise. In New York it has had victims only with the neglected foreign population, whose habits and hygienic conditions would breed, in warm weather, cholera, plague, or some other fatal scourge by a fixed law of nature—that *mammals* are not made to live in all elements—to breathe water, air, and the most noxious gases conceivable, and continue for any great length of time in healthy condition.

We have from the first endeavored to show that cholera is not contagious, and that those only are greatly exposed to attack who are intemperate and imprudent, whose habits and manner of life induce the disease. If, however, this faith shall be shown unfounded we shall cheerfully abandon it and take refuge in any better established truth. We propose to avoid, and advise others to avoid positive conclusions concerning this disease, hoping still to be able to arrive at more positive knowledge; meanwhile to look upon the most cheerful side of the question and avoid as far as possible all conclusions which if adopted, would produce injury and alarm.

An experienced medical friend, whose observation extends over the whole period since the first outbreak of cholera in this country, and whose judgment and practical ability entitle his opinions to the highest respect, assured us this morning that the first appearance of cholera marked a period of great change in diseases of all forms, that pneumonia was never afterwards so sthenic in its type, pleurisy was less acutely inflammatory, and was more often attended by a low form of constitutional fever. That inflammations were not so active in their symptoms and progress, and that the prevailing autumnal and typhoid fevers were of a much lower grade. Without advocating any change of type theory, with the object of shielding the profession from charge of unnecessary and unjustifiable activity in treatment during the *age of depletion*, he still maintains that all disease has been modified by causes which are not understood, and is not since the first appearance of cholera in this country, so active and inflammatory as it was before that time; that this is true of disease in districts where cholera was never prevalent, in the rural and mountainous regions, that everywhere a change was obvious, and that a depressing influence, a tendency to low forms of disease was manifest. This is an opinion which is often expressed by physicians of the highest attainment, whose views are worthy of the most careful consideration. We have stated this argument not to adopt or oppose it, but as a succinct statement of the opinions of some men eminently qualified to judge, hoping at least, it may be instructive to the younger members of the profession whose opportunities to know of the opinions and practices of the medical fathers, is confined to history alone. We confess to a sympathy with these view in some respects, and with a few explanations, while in others believe these physicians like the young sailor, who thought the ship stood still, and that the land was moving. We are willing to compromise and conclude that both the ship and land—the knowledge of disease and manner of treating it—are moving, that both therapeutics and disease have undergone change. Certainly we have new diseases and new forms of the same disease.

---

Prof. Wm. Warren Greene, of Pittsfield, Mass., on July 18th, removed a bronchocele from the neck of a lady aged 44 years, which is believed to be the largest ever successfully operated upon. A full report will soon be made of the case.

